# Surgical treatment of rare case of scapula osteochondroma in a resource limited setting: A case report

**DOI:** 10.1016/j.ijscr.2019.07.015

**Published:** 2019-07-22

**Authors:** F.O. Ngongang, G. Fodjeu, A.C. Fon, L. Fonkoue, M.L. Guifo, L.J. Bitang A. Mafok, F. Ibrahima

**Affiliations:** a Department of Surgery and Specialties, Université des Montagnes, Bangangté, Cameroon; b Department of Surgery and Specialties, Faculty of Medicine and Biomedical Sciences, University of Yaoundé 1, Yaoundé, Cameroon; c Yaoundé Emergency Center, Yaoundé, Cameroon; d Faculty of Medicine, University of Ngaoundere, Cameroon

**Keywords:** Osteochondroma, Scapula, Surgical, Case report

## Abstract

**Introduction:**

Osteochondroma also known as exostosis is one of the most common benign bone tumours, and are characterized by bone protuberances surrounded by a cartilage layer. They generally affect the extremities of the long bones in an immature skeleton and deform them. They are easily diagnosed at the level of the appendicular skeleton. However, atypical localization and malignancy are sometimes challenging to diagnose on clinical evaluation and plain radiography. Therefore, more refine diagnostic tools may be required. Scapula localization of solitary exostosis is relatively rare.

**Case presentation:**

We report the case of a 17 years old male patient reporting worsening shoulder pain since 3 years with right scapula winging. Computer tomography (CT) scan revealed an osteochondroma on the ventro-medial surface of the right scapula extending into the scapulo-thoracic space. Surgical excision was done and histopathological study confirmed osteochondroma of the scapula. We noted excellent post-operative pain alleviation after two weeks, full range of motion and a better self-esteem.

**Discussion:**

The scapula is involved in 3.0–6.4% of all cases. Osteochondroma usually arises on the anterior surface of the scapula. Surgical excision is an excellent treatment option for symptomatic patients with scapula osteochondroma. In our case presentation we used a muscle sparing technique. No muscle detachment will ensure rapid and better postoperative recovery. Surgical removal is useful in eliminating painful symptoms and avoids possible malignant transformation.

**Conclusion:**

Good clinical outcome is expected with surgical excision of symptomatic scapula osteochondromas especially using muscle-sparing technique which offers a quick functional rehabilitation of patients.

## Highlights

• Osteochondroma is a frequent benign tumor in the growing adolescent and involves mostly the growth plate of long bones and seldomly the scapula.
• Solitary osteochondroma of the scapula is a rare and usually incidental finding and usually asymptomatic, but for its compressive effect that can be at the origin of pain, winging, snapping noise, limited range of motion and even possible fractures.
• The therapeutic options of this rare finding are not clearly codified in our settings. Thus, we present a case of a solitary scapular osteochondroma with pressure symptoms managed surgically.

## 1 Introduction

Osteochondromas or exostoses (external bone proliferation that deforms the bone) are the most common benign tumours of bone. They account for 35%–46% of all benign neoplasms of bone [1]. This bone protuberance is generally found in the immature skeleton of children and adolescents, and their growth usually ceases when skeletal maturity is reached [2]. According to the World Health Organization (WHO), osteochondromas are bone projections enveloped by a cartilage cover that arise on the external surface of the bone [3]. Despite their predominant composition of bone, their growth is via progressive endochondral ossification of the cartilaginous cap. They present two distinct clinical forms: developing in the metaphyseal region of long bones either alone or in connection with the hereditary multiple exostoses syndrome, an autosomal dominant disorder characterized by the formation of multiple cartilaginous osteochondromata in the immature skeleton [4]. About 90% are solitary exostoses and may occur on any bone but usually found on the metaphysis of long bones [2]. Osteochondroma comprises of about 35%–46% of all benign bone tumours [1]. About 90% occur in the metaphysis of tibia, humerus and distal femur [2,9]. The scapula is involved in 3.0–6.4% of all cases.

These tumours are usually asymptotic and are discovered incidentally. Some patents may present with pain due to mechanical pressure to surrounding structures, fracture of the bony stalk of the tumour, neurovascular impingement, bursa formation and rarely malignant transformation of the cartilaginous cap, and only then is surgery considered best treatment [5,6].

Literature on the surgical technique of excision of symptomatic exostosis is limited [4,7]. We therefore present the case of a 17 years old patient with symptomatic ventro-medial right scapula solitary exostosis. This case was reported in line with the SCARE criteria [8].

## 2 Case presentation

We report the case of 17 years old right-handed male who presented in our outpatient department with progressive right shoulder pain for 02 years. During the last 01 year he developed gradual right scapula winging with limitation of overhead activities. There was no notion of trauma or fever. Patient was otherwise healthy with no pertinent family history. He had several consultations with an attempted excision during one of his previous consultations by an inexperienced health personnel.

**Fig. 1:**
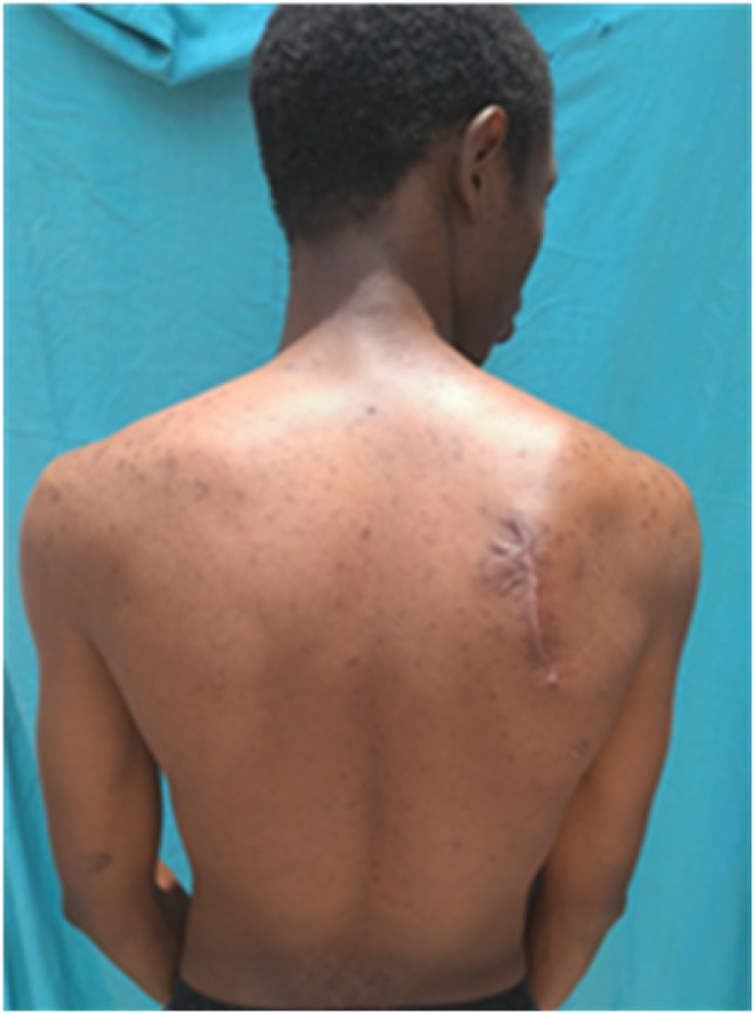
Attempted surgical scar and right scapula winging.

**Fig. 2:**
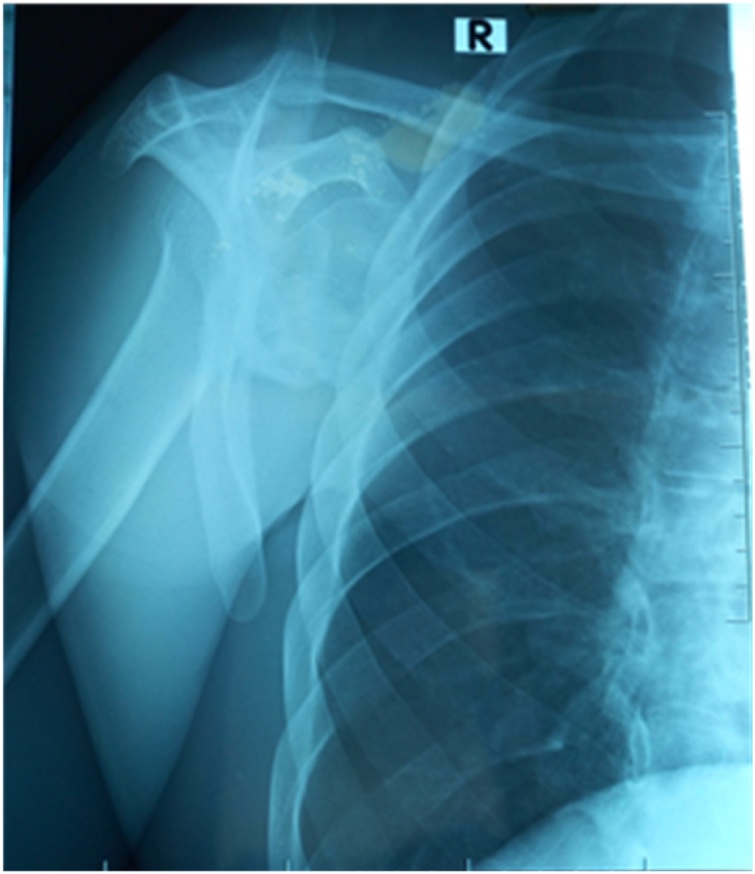
Lateral shoulder view showing a right subscapular mass.

**Fig. 3:**
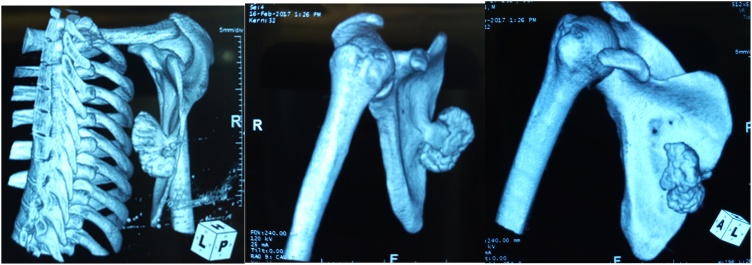
CT scan 3D reconstruction of the right scapula showing exostosis.

Physical examination showed an asymmetry of his scapulae with a wing-like prominence of his right scapula giving a right medial scapula elevation from thoracic cage of about 4 cm and a difficultly palpable mass with crepitus of the shoulder on mobilization (Fig. 1). Elsewhere on inspection, we had a longitudinal scar of about 7 cm on the medial border of the right scapula from an attempted excision by inexperienced medical personnel (Fig. 1). A full range of motion was found in both shoulders. Radiographic evaluation showed an irregular bony structure extruding from the scapula (Fig. 2). Computed tomography (CT) revealed a bony exostosis along the medial border on the ventral surface of the right scapula (Fig. 3). There were no signs of malignant transformation.

## 3 Surgical procedure

**Fig. 4:**
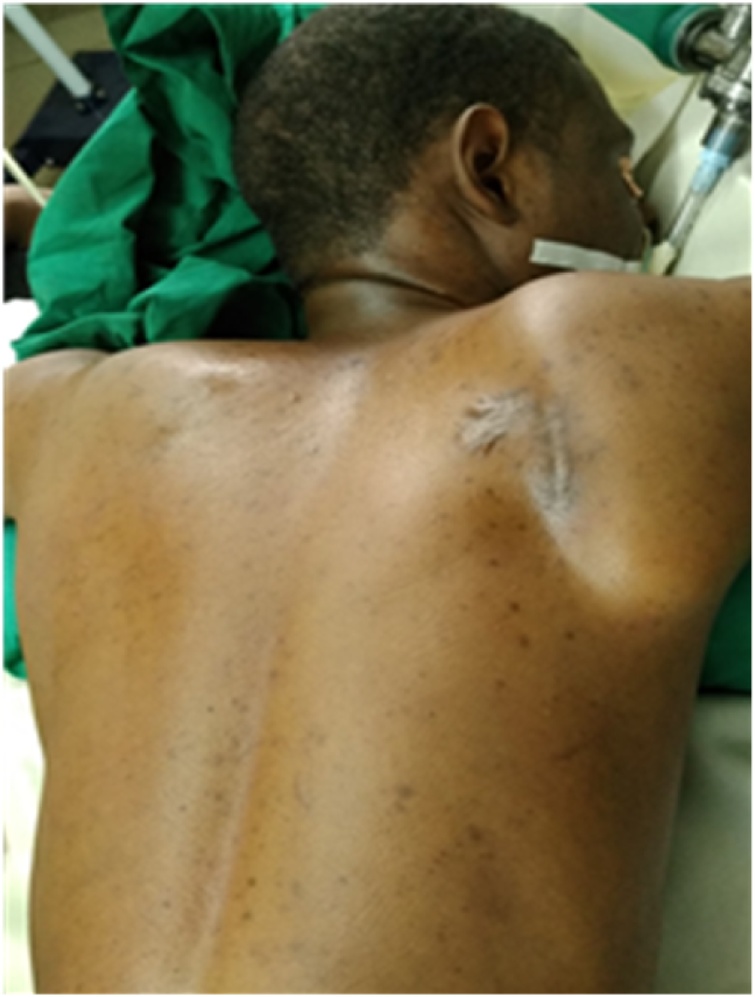
Patient positioning.

**Fig. 5:**
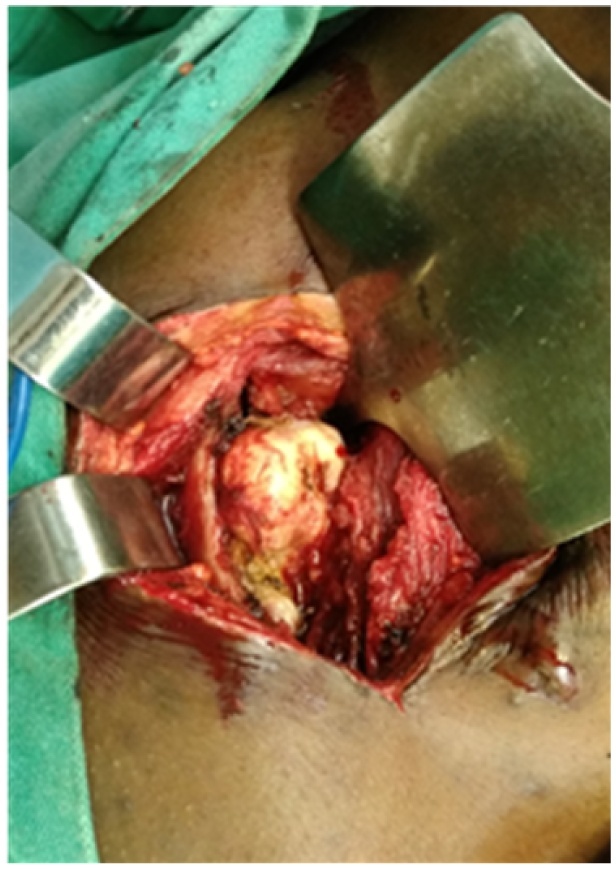
Exposure of exostosis.

**Fig. 6:**
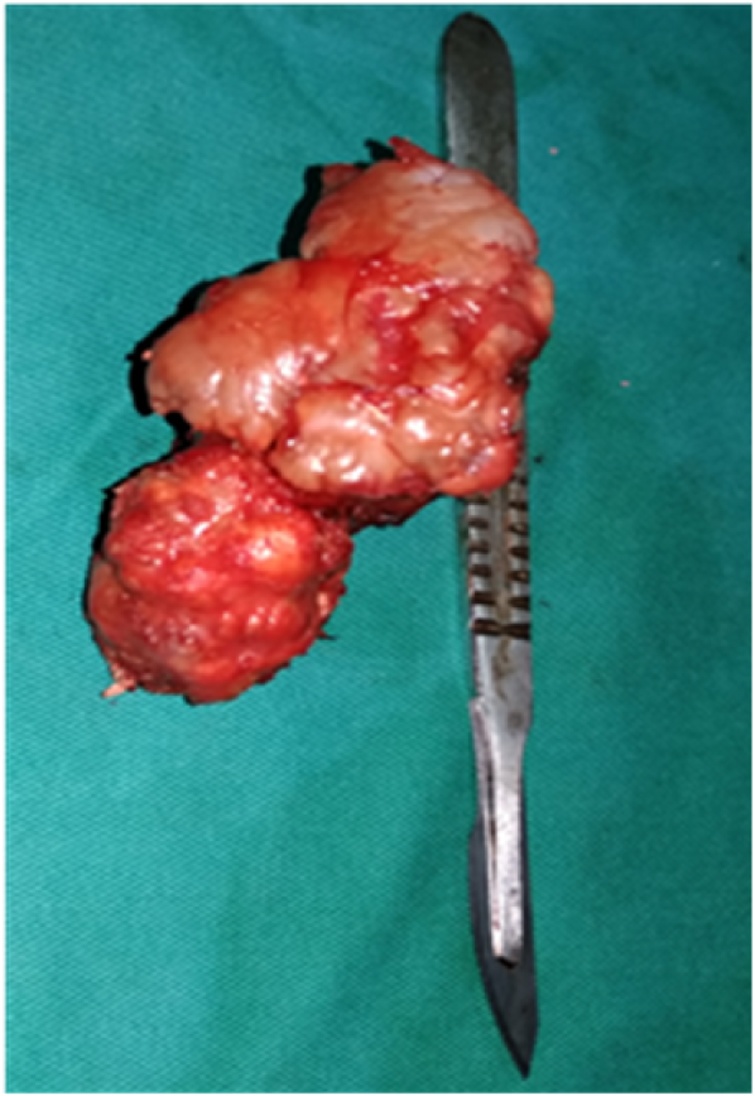
Exostosis once resected.

**Fig. 7:**
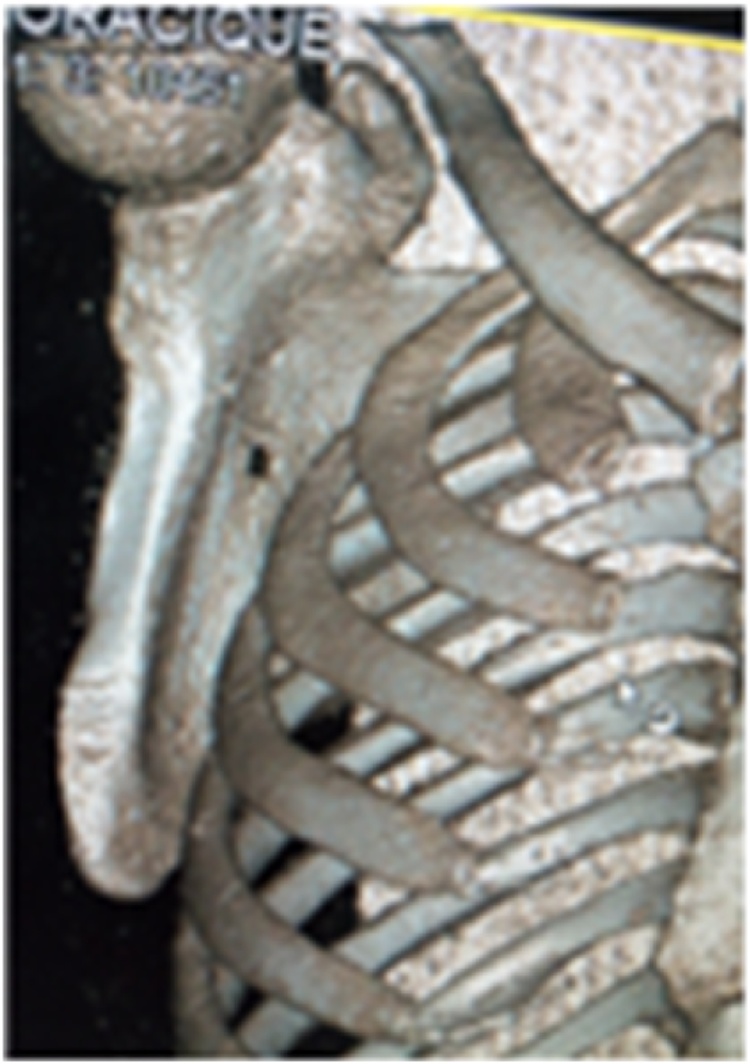
Scapula after excision of osteochondroma.

**Fig. 8:**
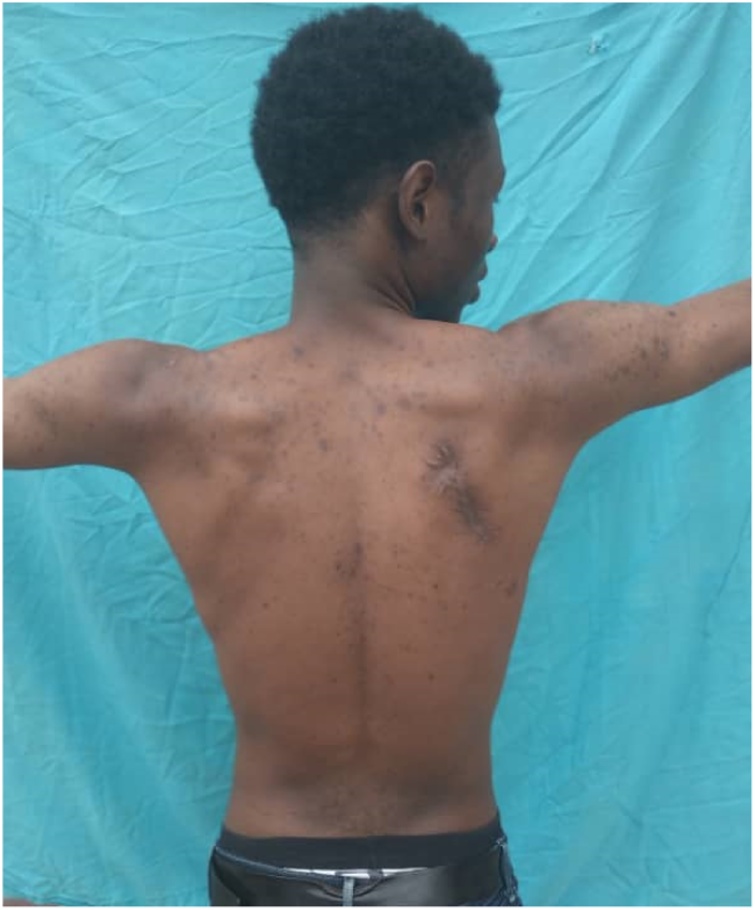
Right scapula abduction at 1 year.

Patient was placed in a prone position under general anesthesia (Fig. 4). A parascapular incision was made along the medial border of the right scapula. Sharp dissection was carried out down to the level of the fascia of the trapezius muscle. After opening the fascia the trapezius muscle was retracted following its fibers cranially. The rhomboid was split bluntly in line with the fibers and subperiosteal dissection allowed full exposure of the exostosis (Fig. 5). The stalk of the exostosis was excised at the base with an osteotome from the ventral surface of the scapula. The specimen measured 9 cm × 5 cm (Fig. 6). The different muscular fibers layers fell back against each other after removal of the exostosis. A Redon vacuum drain was placed followed by the closure of the trapezius fascia and finally, the wound was closed in layers. Histologic examination confirmed that the specimen was an osteochondroma with no signs of malignant transformation. Patient was placed in a sling for pain relief for one week. Immediate range of motion was started as tolerated by the patient. Pain relief was excellent; there was no crepitus and scapula deformity. By three weeks full range of motion was possible without pain or discomfort (Fig. 7). Patient had no pain, full range of right shoulder motion without discomfort at one year follow up (Fig. 8)

## 4 Discussion

Clinical manifestation of osteochondroma of the scapula is strictly correlated to its size and location. Symptoms result from mechanical irritation of muscle, tendon or soft tissue, formation of a pseudo aneurysm or bursa, fracture, or malignant transformation [9,10]. Osteochondroma of the scapula usually arises on its anterior surface (was it similar to our own?) [11,12]. Surgical excision is an excellent treatment approach for symptomatic patients with scapula exostosis. There are 3 main surgical approaches to removal of scapula exostosis described in literature: muscles sparing, muscle detaching and endoscopically assisted techniques [4,7]. In our case presentation we used a muscle sparing technique. No muscle detachment will ensure less blood loss, rapid and better postoperative recovery. This recovery time maybe even much shorter with endoscopy techniques alongside a better cosmetic outcome [4,7,9]. Giving the limited access to endoscopic techniques in our resource limited settings, muscle sparing technique is a better alternative with good results. Surgical removal is useful in eliminating painful symptoms, discomfort and avoids possible malignant transformation.

## 5 Conclusion

The diagnosis osteochondroma of the scapula should be considered in any patient with scapular pseudo-winging, crepitus and pain of shoulder region between 10–30 years of age. Good clinical outcome can be expected with surgical excision of symptomatic ventral osteochondromas of the scapula. Muscle-sparing technique offers a quick functional rehabilitation of patients with symptomatic osteochondromas in resource limited settings.

## Sources of funding

This research did not receive any funding.

## Ethical approval

This study was performed in accordance with the guidelines of the Helsinki Declaration and was approved by the Ethical board of the faculty of medicine and biomedical science of the University of Yaoundé 1.

## Consent

Written informed consent was obtained from the patient’s parents for publication of this case report and accompanying images.

## Author contribution

Conceptualization, Methodology: **NFO, FG, FAC** Data curation, Writing- Original draft preparation: **NFO, FG, FAC** Statistical analysis and interpretation **NFO, FG**. Drafting: **NFO, FG, FAC, GML, FL**. Critical discussion and manuscript revision: **NFO, FG, CFA, GML, FL, BAM, IF.** Supervision**: IF.** All authors read and approved the final manuscript.

## Registration of research studies

N/A.

## Guarantor

NGONGANG Frank Olivier.

## Availability of data and materials

Data will be available from the corresponding author upon request.

## Provenance and peer review

Not commissioned, externally peer-reviewed.

## Declaration of Competing Interest

None declared. The authors have no financial, consultative, institutional, and other relationships that might lead to bias or conflict of interest.
